# Proteomic risk scores for predicting common diseases using linear and neural network models in the UK biobank

**DOI:** 10.1038/s41598-025-06232-1

**Published:** 2025-07-01

**Authors:** Alexander Smith, Paul Elliott, Manuel Mayr, Abbas Dehghan, Ioanna Tzoulaki

**Affiliations:** 1 Department of Epidemiology and Biostatistics, School of Public Health, Imperial College London, London, UK; 2 UK Dementia Research Institute at Imperial College London, Hammersmith Hospital, London, UK; 3 National Heart & Lung Institute, Imperial College London, London, UK; 4 Centre for Systems Biology, Biomedical Research Foundation of the Academy of Athens, Athens, Greece; 5 Department of Epidemiology and Biostatistics, Sir Michael Uren Hub, 86 Wood Lane, London, W12 0BZ UK

**Keywords:** Proteomics, Machine learning, Predictive medicine

## Abstract

Plasma proteomics provides a unique opportunity to enhance disease prediction by capturing protein expression patterns linked to diverse pathological processes. Leveraging data from 2,923 proteins measured in 53,030 UK Biobank participants, we developed proteomic risk scores for 27 common outcomes over 5- and 15-year follow-up periods using two approaches: a linear ElasticNet regression model and a deep learning neural network (NN) model. Using Cox regression, we assessed the discrimination of proteomic risk scores either in isolation or as incremental improvements over clinical risk factors. We also studied the shared and unique protein predictors across conditions. Proteomic risk scores demonstrated strong discrimination for most outcomes, with a C-index > 0.80 for 12 diseases. NN models outperformed linear models for 11 outcomes, particularly for diseases such as Parkinson’s disease (C-index 0.84) and pulmonary embolism (C-index 0.83), where nonlinear relationships contributed significantly to prediction. Across all outcomes, the addition of proteomic scores to clinical models improved predictive accuracy (ΔC-index 0.03), with the greatest gains observed in 9 diseases (ΔC-index > 0.1), including end-stage renal disease, pulmonary embolism, and Parkinson’s disease. Analysis of protein contributions revealed shared predictors across multiple diseases, such as growth differentiation factor 15 (GDF15), as well as unique predictors like PAEP for endometriosis. While NN models may capture complex relationships, linear models provided value through simplicity and interpretability. These findings underscore the importance of tailoring predictive approaches to specific diseases and demonstrate the pivotal potential of proteomics in advancing risk stratification and early detection.

**Supplementary Information:**

The online version contains supplementary material available at 10.1038/s41598-025-06232-1.

## Introduction

Plasma proteomics, the simultaneous detection and quantification of thousands of plasma proteins, offers a unique window into protein expression patterns that may reflect disease pathophysiology^1^. Although proteins are often studied to enhance our understanding of disease biological underpinnings, this information may also provide powerful tools for risk stratification purposes. The comprehensive and unbiased nature of the technique offers a wealth of information which can be combined into disease specific proteomic risk scores to estimate an individual’s risk of developing different conditions. The ease and the relatively low cost of measurement constitute further attractive features of proteins as predictive risk factors.

Prediction of future disease has never been more important as globally more people are living with one or more conditions, and preventive treatment and screening options are increasing. Identification of high-risk individuals is important to guide clinical decisions, healthcare policy and risk communication^2^. Several proteins are already incorporated into disease risk stratification models such as measurements of lipids for Coronary Heart Disease (CHD)^3,4^ prediction or Prostate Specific Antigen (PSA) antigen for prostate cancer screening guidance^5^. More recently, the availability of large-scale proteomic data in large epidemiological studies has highlighted the potential of several linear proteomic risk scores to predict common and rare disease outcomes^6,7^.

Here, we also utilized the available proteomics measurement of 2,923 proteins in 53,030 UK Biobank participants to further study different definitions of proteomic risk scores to predict long- and short-term incidence of several common diseases. We introduce a novel approach to constructing proteomic risk scores using deep learning neural networks (NN). Unlike traditional regression-based methods, NN can capture nonlinear relationships and complex interactions between proteins, offering a more comprehensive and nuanced representation of the proteomic landscape. We hypothesised that applied to proteomic data, these models may identify subtle proteomic signatures that may otherwise be overlooked by linear methods, potentially improving the predictive accuracy of risk scores for chronic diseases. We then compared the predictive value of the NN proteomic risk scores to the simpler linear ones as well as to clinical risk predictors and assessed their incremental improvement for disease prediction. Finally, we examined which proteins are important for different disease outcomes and highlighted proteins which are common or unique predictors of different diseases.

## Results

**Fig. 1:**
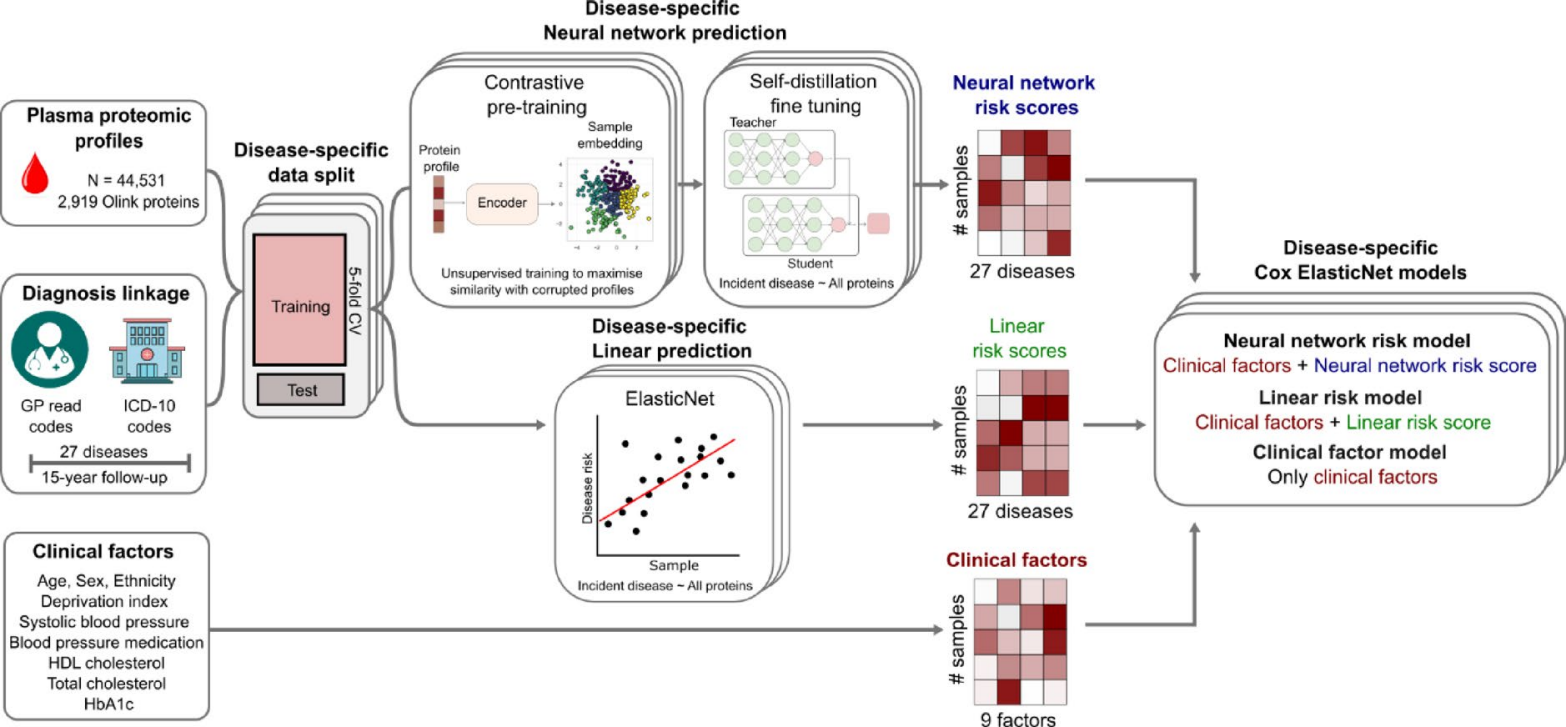
Schematic diagram of the study design.

A schematic of the overall study is given in Fig. 1. We derived proteomic risk scores in UK Biobank using a linear ElasticNet regression model (linear model) and a non-linear deep learning NN model in the same training and test set splits on a per outcome basis to allow for accurate comparison, over two prediction horizons (5 and 15 years). We compared the predictive accuracy of the two methods of proteomic risk scores and studied their incremental performance to a simple clinical model (Methods).

We analysed 53,030 UK Biobank participants (Supplementary Table 1 list the main baseline characteristics of the UKB study samples used in this study) and 27 different outcomes with more than 100 incident events based on 15-year follow-up (range from 4,070 (CHD) to 119 (schizophrenia); Table 1).

**Table 1:** Summary of the incident disease diagnoses within UK biobank cohort across two different follow up periods. Median follow-up time is based on the follow-up time from proteomic plasma measurement (baseline) until time to event (first diagnosis, death or censoring date, whichever occurs first).

		15 years follow-up		5 years follow up	
Condition	Prevalent cases (*N*)	Cases (*N*)	Mean follow-up time (SD)	Cases (*N*)	Mean follow-up time (SD)
Parkinson’s disease	42	430	8.67 (4.25)	102	2.81 (1.37)
Atrial fibrillation	619	3342	9.08 (4.24)	707	2.82 (1.43)
Asthma	2225	2849	7.43 (4.26)	951	2.64 (1.41)
COPD	381	2394	8.48 (4.22)	596	2.80 (1.39)
Dementia	10	1096	10.50 (3.59)	98	3.59 (1.23)
Depression	1034	2662	8.80 (4.05)	554	2.87 (1.45)
Type-2 Diabetes	646	2332	9.06 (4.11)	453	2.78 (1.44)
Endometriosis	298	184	6.28 (4.20)	85	2.57 (1.50)
Primary Malignancy – Pancreatic	4	185	9.68 (4.01)	34	3.35 (1.26)
Heart failure	247	2015	9.55 (4.29)	388	2.76 (1.45)
Motor neuron disease	29	288	7.19 (3.19)	81	3.31 (1.20)
Multiple sclerosis	226	167	7.25 (4.40)	61	2.58 (1.51)
Pulmonary embolism	163	895	9.33 (4.18)	162	2.89 (1.44)
Primary pulmonary hypertension	115	369	5.10 (3.13)	221	3.06 (1.39)
Primary Malignancy - Colorectal and anus	155	776	8.86 (4.38)	184	2.67 (1.40)
Primary Malignancy - Brain, Other CNS and Intracranial	12	125	8.25 (4.06)	36	3.33 (1.28)
Primary Malignancy – Breast	659	1217	8.04 (4.39)	359	2.71 (1.43)
Primary Malignancy - Lung and trachea	32	615	9.16 (4.10)	137	3.35 (1.28)
Primary Malignancy – Prostate	131	1268	9.04 (4.36)	287	2.73 (1.46)
Rheumatoid Arthritis	186	664	9.54 (3.66)	80	3.20 (1.47)
Schizophrenia	99	119	8.38 (4.30)	28	2.54 (1.72)
Lupus erythematosus (local and systemic)	267	259	5.84 (3.48)	116	2.68 (1.36)
Psoriasis	474	476	7.89 (4.68)	156	2.38 (1.36)
Primary Malignancy – Gynaecological	126	332	7.98 (4.33)	107	2.88 (1.41)
Coronary heart disease	1296	4070	8.24 (4.39)	1137	2.63 (1.45)
Ischaemic stroke	93	921	9.79 (4.05)	148	2.98 (1.40)
End stage renal disease	39	177	9.39 (4.00)	28	2.88 (1.08)

COPD: Chronic obstructive pulmonary disease

### Linear vs. Neural network proteomic risk scores

**Fig. 2:**
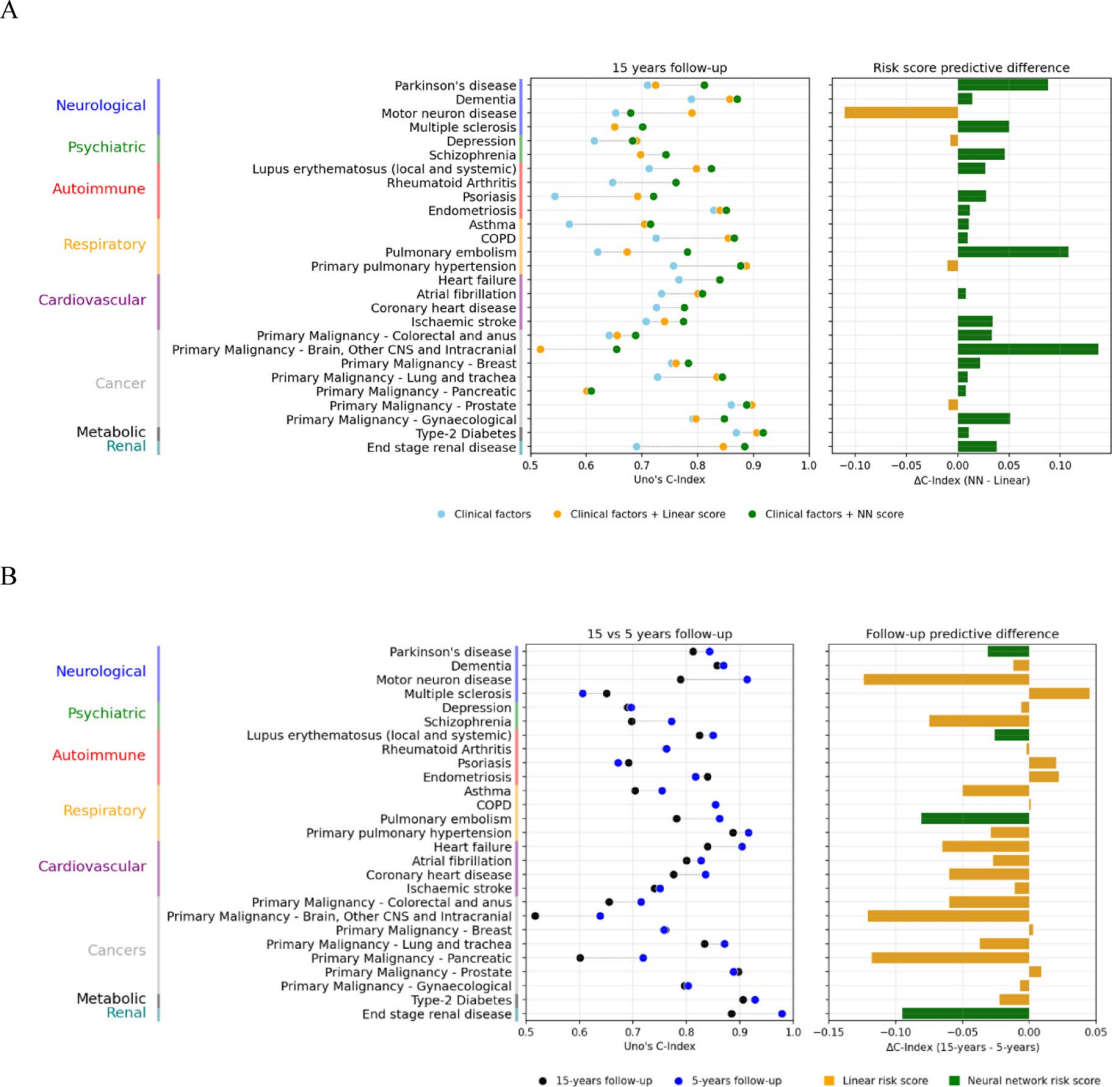
(**A**) C-index across examined diseases for 15-years follow-up in survival analysis of UK Biobank participants. Models with yellow bars in the predictive difference plot indicate outcomes where the linear risk score outperforms the neural network risk score. (**B**) Comparison of the C-index discriminatory performance of a survival model containing both clinical risk factors and a protein risk score for 15 years and 5 years of follow-up. Both follow-up periods use the same risk scores (linear or neural network) within each disease. Models with yellow bars in the predictive difference plot indicate models which use a linear risk score, whilst green bars use a neural network risk score (Parkinson’s disease, lupus erythematosus, pulmonary embolism and end-stage renal disease).

Proteomic profile derived disease risk scores were generated for 27 outcomes using the proteomics assay of 2,919 proteins. Linear and NN proteomic risk scores were equivalent (define as their difference in C (ΔC) index < 0.02) for only half of examined outcomes (*N* = 14) (Supplementary Tables 3and Fig. 2A). The NN proteomic risk score outperformed the linear score (Fig. 2A) for 11 outcomes with the highest difference seen for multiple sclerosis (ΔC index difference = 0.20) for 15 years prediction horizon. Similar results were seen for the 5 years prediction, where the overall discrimination of proteomic risk scores for most outcomes was higher (Supplementary Table 4).

Overall, protein risk scores achieved very good discrimination for most diseases with C index > 0.75 for 19 outcomes and > 0.80 for 12 outcomes. The highest discriminatory performance for 15 years was seen for end stage renal disease (NN proteomic risk score C index = 0.89 for 15 years and 0.98 for 5 years).

## Proteomic risk scores and clinical factors over 15 years of follow up

The baseline performance of a clinical model based on standard cardiometabolic risk factors achieved good discrimination (C index > 0.70) for 16 different outcomes including non-cardiovascular outcomes such as prostate cancer, endometriosis, and lupus erythematosus (Supplementary Table 3, Fig. 2A). However, when compared to a model with just age and sex, clinical risk factors had ΔC index > 0.02 for 12 outcomes with the largest increases seen for type-2 diabetes (ΔC index = 0.25), schizophrenia (0.20), lupus erythematosus (0.07) and depression (0.07).

We then evaluated the discrimination of proteomic risk scores compared to the baseline model of standard clinical risk factors (Fig. 2A). Due to the simplicity of the linear proteomic score, we opted for the linear score when the performance between the two was equivalent or greater than the NN equivalent (*N* = 16 outcomes with linear scores, *N* = 11 outcomes with NN scores). The proteomic risk scores performed better than models using only the clinical factors (ΔC index difference > 0.02) for 21 out of the 27 examined outcomes (average C index difference 0.03 across all outcomes). Overall, there were 9 outcomes (end stage renal disease, pulmonary embolism, COPD, primary pulmonary hypertension, lung and trachea cancer, rheumatoid arthritis, lupus erythematosus, motor neuron disease and Parkinson’s disease) which had a combined C index (clinical and proteomic risk score) > 0.75 indicating good discrimination and where the addition of the protein risk score increased the C index > 0.1 compared to the clinical model indicating incremental value of the proteins in risk prediction. Of them, the improvement for Parkinson’s disease, end stage renal disease, lupus erythematosus and pulmonary embolism was seen via the NN proteomic risk score.

## Proteomic risk score for 5 years vs. 15 years of follow up

Overall, when comparing the performance improvement due to the addition of a protein risk score to a model containing only clinical risk factors, the results were similar for the 5-year time horizon, although the proteomic risk scores showed higher discrimination than their 15 years equivalent (Fig. 2B). We identified 9 outcomes where the addition of a protein risk score to a clinical factor model improved the 5-year prediction by greater than 0.1 C-index and an overall C index was greater than 0.75 (asthma, COPD, heart failure, motor neuron disease, pulmonary embolism, primary pulmonary hypertension, lung and trachea cancer, and rheumatoid arthritis).

## Protein contributions to disease risk scores

We investigated the proteins contributing to the linear and NN risk scores. Linear risk scores are much more interpretable than the complex non-linear NN models. Therefore, we investigated the proteins contributing to the linear risk scores by inspecting the beta coefficients of each ElasticNet regressions for the 20 outcomes which improved upon addition of the linear risk score but were not outperformed (ΔC index < 0.05) by the non-linear risk score (Supplementary Table 3).

**Fig. 3:**
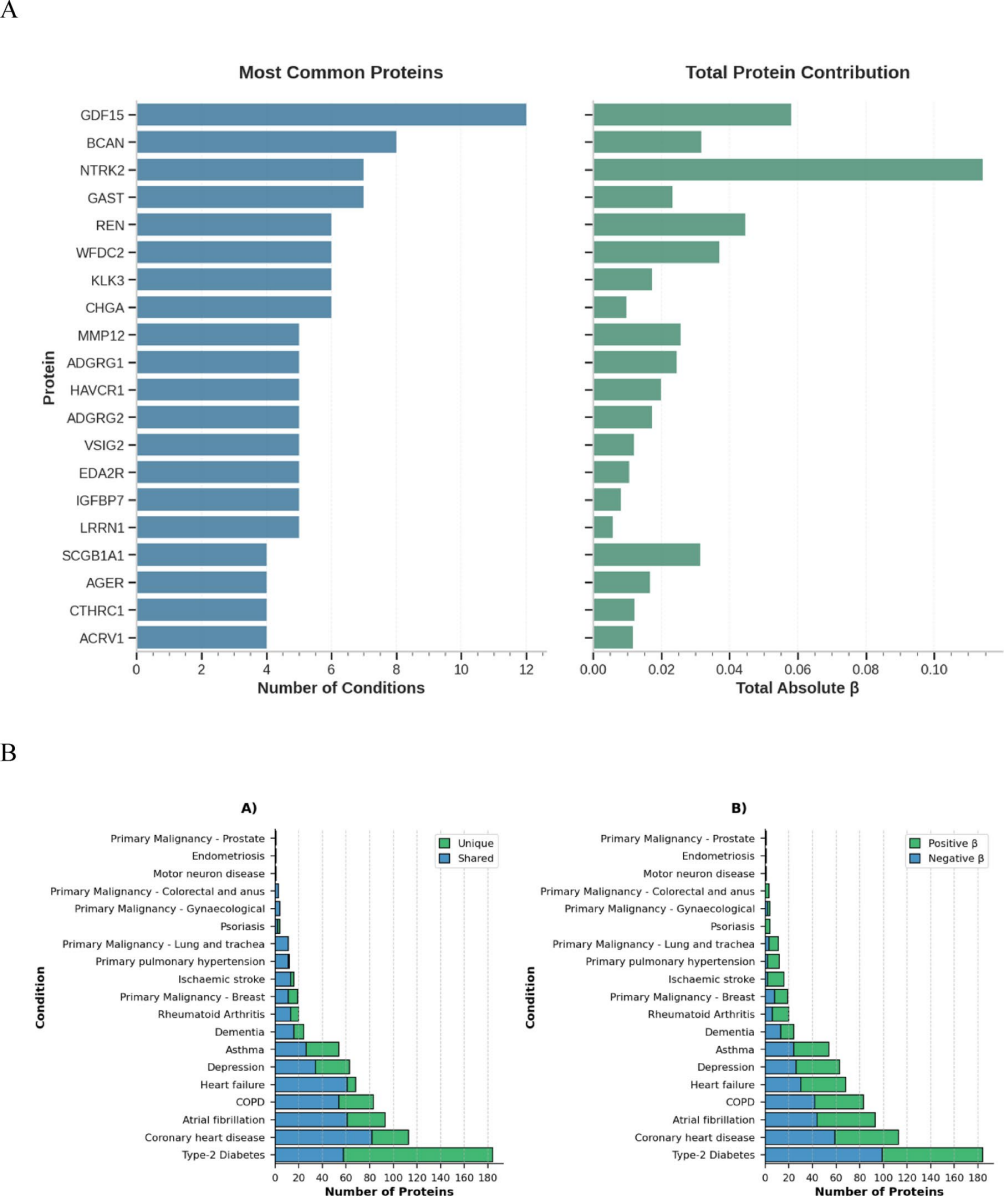
(**A**) The number of diseases each protein contributes to and the sum of their absolute beta co-efficient across all linear risk scores, restricted to the top 20 proteins. (**B**) The number of protein’s contributing to each of the linear disease risk scores which are either unique to the condition or shared across multiple conditions, and the number of proteins with a positive or negative contribution.

**Fig. 4:**
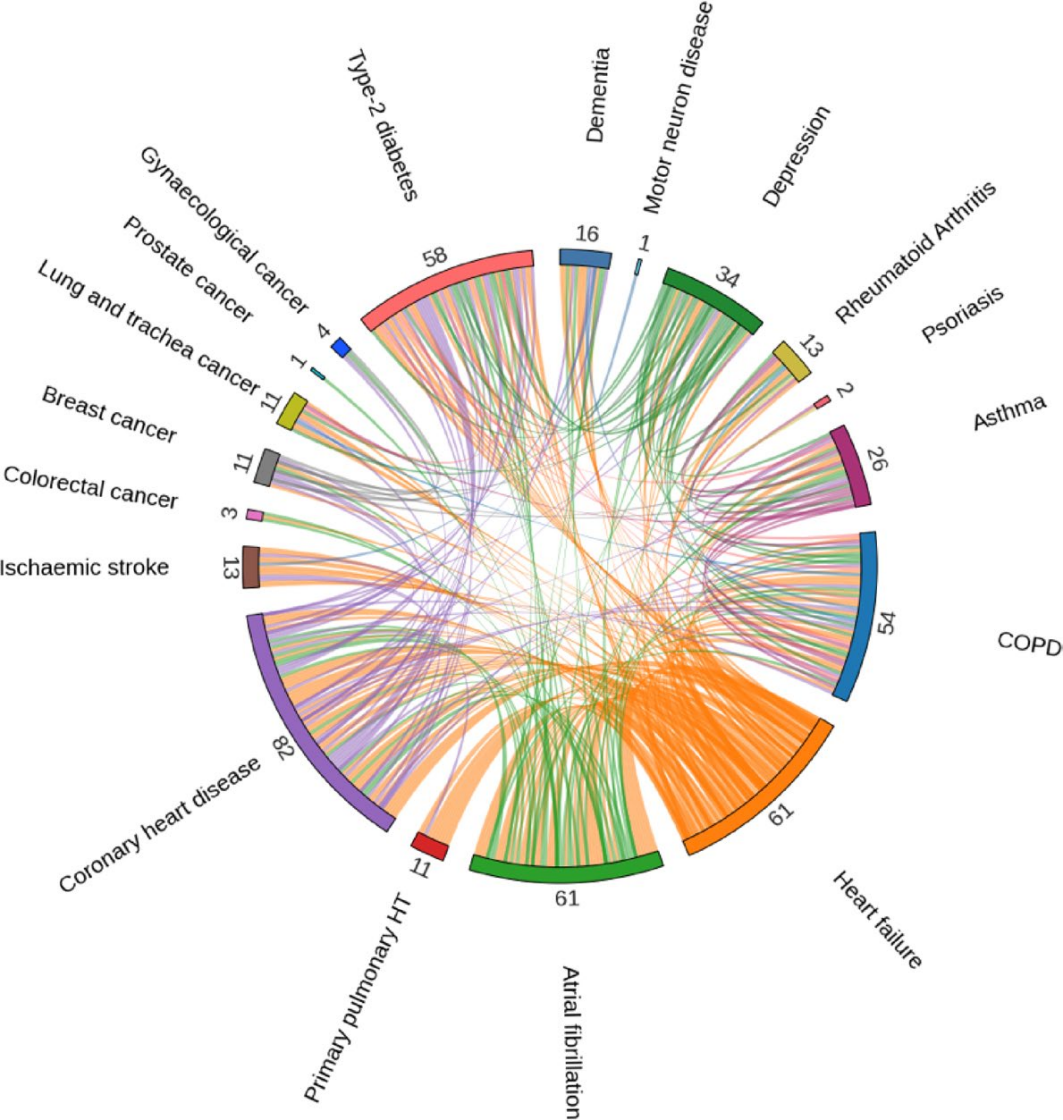
Overlap of the shared proteins contributing to each of the linear risk scores.

Out of the assay of 2,919 proteins included in the generation of every linear risk score, only 474 proteins contributed to at least one risk score and 312 of these were unique to a single disease risk score (see Supplementary Table 5). Growth differentiate factor 15 (GDF15) was the most frequent contributor to the risk scores (12 diseases) (Fig. 3A). The protein with the largest number of inverse relationships were BCAN (8 diseases). Neurotrophic receptor tyrosine kinase 2 (NTRK2) had the largest contribution to risk scores with a total absolute beta of 0.11 across 7 diseases. The number of proteins contributing to each disease risk score varied greatly (Fig. 3B, Supplementary Table 6). Type-2 diabetes had the largest number of protein contributions (184 proteins) whilst three conditions had only one protein generating their risk scores: motor neuron disease (NEFL), prostate cancer (KLK3) and endometriosis (PAEP). Three outcomes had only unique proteins: endometriosis (1 protein), motor neuron disease (1 protein) and psoriasis (4 proteins). The diseases with the largest ratio of unique proteins were ischaemic stroke (14 unique, 2 shared), type-2 diabetes (154 unique, 30 shared) and primary pulmonary hypertension (10 unique, 2 shared). There was a large overlap in proteins contributing to risk scores for cardiovascular outcomes (Fig. 4).

## Non-linear neural network proteomic risk scores

For the 4 outcomes (Parkinson’s disease, end stage renal disease, pulmonary embolism, lupus erythematosus) where the NN risk score outperformed its linear counterpart (ΔC index difference > 0.02) and provided a significant improvement over the clinical factors (ΔC index > 0.1) we examined the contribution of proteins using SHAP values. Across the four outcomes, a total of 878 proteins had non-zero SHAP values in at least 25% of samples and 67 contributed to more than one non-linear NN risk score (see Supplementary Table 7). The number of globally important proteins for each disease risk score ranged from 25 proteins (lupus erythematosus) to 449 (pulmonary embolism).

**Fig. 5:**
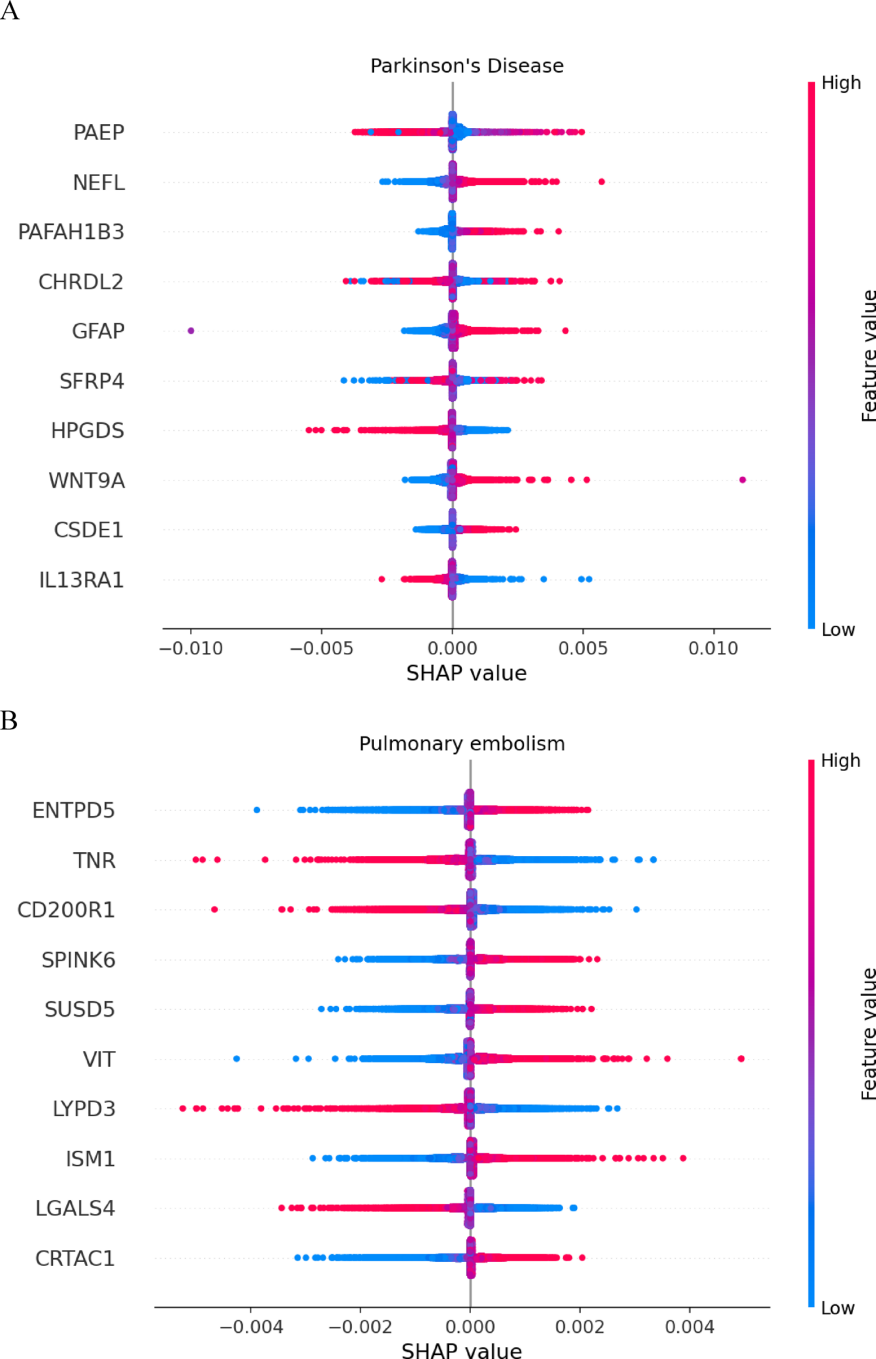
Bee swarm plots for protein SHAP values for Parkinson’s disease (**A**) and pulmonary embolism (**B**).

All four diseases had no single predominant protein responsible for the risk score but instead consisted of a large set of proteins with similar SHAP values (Fig. 5 and Extended Data Fig. 2). For example, in Parkinson’s disease, the top ten proteins had similar overall importance to the generation of the NN risk score based on similar total absolute SHAP values (PAEP, NEFL, PAFAH1B3, CHRDL2, GFAP, SFRP4, HPGDS, WNT9A, CSDE and IL13RA1); HPGDS and IL13RA1 had an inverse association with disease risk (Fig. 5A). The top ten proteins for pulmonary embolism also had similar overall importance to the generation of the NN risk score (ENTPD5, TNR, CD200R1, SPINK6, SUSD5, VIT, LYPD3, ISM1, LGALS4, CRTAC1) and four proteins (TNR, CD200R1, LYPD3 and CRTAC1) had an inverse association with disease risk (Fig. 5B). Results for lupus erythematosus and end stage renal disease are shown in Extended Data Fig. 2.

## Discussion

In this study, we demonstrated that plasma proteomics, through the generation of proteomic risk scores, offers significant potential not only for understanding disease pathophysiology but also as a powerful tool for predicting both short- and long-term risk of various common diseases. Our findings show that state of the art deep learning neural network (NN)-based proteomic risk scores often outperform their linear counterparts and highlight several diseases where the added value of proteomic data enhances established clinical risk factors, achieving high model discrimination (C-index > 0.75). Furthermore, we identified several proteins that are consistently included in risk scores across multiple diseases, suggesting their broader relevance in predicting multimorbidity but also proteins that are unique to certain diseases. Overall, our results underscore the new possibilities of proteomic risk scores for targeted screening and early prevention, particularly for diseases like Parkinson’s disease and multiple sclerosis, which lack effective early detection strategies, as well as for predicting the risk of multiple conditions simultaneously.

The comparative performance between linear and non-linear NN risk scores across all diseases showed that non-linear risk scores generally outperformed linear models in both follow-up periods with marked differences for certain diseases such as Parkinson’s disease and motor neuron disease. Unlike linear models, which assume direct and additive relationships between proteins and disease risk, NN models can capture complex non-linear effects within the proteomic data that may otherwise be overlooked while also offering very effective regularization to mitigate the risk of overfitting^8^. This ability likely contributed to improved predictive accuracy. Nevertheless, linear risk scores have the advantage of simplicity and interpretability, making them preferable when their predictive performance is comparable to that of NN models. Our comprehensive approach examining different methodologies for protein risk score development performed better or equally for most of the diseases previously examined in the same UK Biobank population^6^. This emphasises the advantage and robustness of our approach as it demonstrates the absence of a universally optimal risk score and the need for a flexible approach to disease prediction—one that integrates multiple methods to explore the full range of potential solutions and tailor the strategy to specific diseases.

NN proteomic scores showed promising predictive ability for Parkinson’s disease, a disease where there is unmet clinical need to identify high risk individuals before significant neuronal loss and disabling motor and/or cognitive disease. Our proteomic risk score combined with a simple clinical model achieved high discrimination (C index = 0.80) to identify high risk of the disease as early as the 15 years prior to the disease incidence and was even higher (C index = 0.84) for 5 years follow up confirming similar results from other studies using related approaches on proteomics risk scores^9^. This approach which relies solely on easily accessible predictors is particularly advantageous for Parkinson’s disease where risk prediction through invasive lumbar puncture or demanding imaging protocols have been alternatively suggested^10^. The top contributing proteins to the model include proteins implicated in related disease pathways and comorbidities such as GFAP (astroglia activation), NEFL (neuroaxonal damage), IL13RA1 and CHRDL2 (inflammation), SFRP4 and CHRDL2 (bone and joint function) and provide further evidence into the importance of those pathways in identifying high risk individuals or prodromal disease stages. Pulmonary embolism is a life-threatening event whose early prediction can optimize care by enabling targeted surveillance, timely diagnostic imaging, and preventative interventions^11^. Here, we show that a NN proteomics model achieved high discrimination for pulmonary embolism events in a general population over a long 15-year time horizon. Proteins contributing to the risk are involved in processes relevant to disease pathogenesis, including coagulation (VIT, SPINK6), endothelial function (ENTPD5, ISM1, LYPD3), inflammation (LGALS4), and extracellular matrix remodelling (TNR). Previous efforts in have largely focused on risk prediction in individuals with established disease such as venous thromboembolism^12^. There are also several models with simple clinical variables derived from electronic health record data which predict risk of future venous thromboembolism which often precedes pulmonary embolism^13^. Our work highlights the potential utility of proteomic risk scores in capturing complex, multidimensional biological signatures associated with pulmonary embolism events as well as their potential utility to identify patients at risk of severe thrombotic events several years before disease onset and guide prophylactic treatment or help avoid medication which might increase the risk of pulmonary embolism^14,15^.

Pulmonary embolism is a life-threatening event whose early prediction can optimize care by enabling targeted surveillance, timely diagnostic imaging, and preventative interventions^11^. Here, we show that a NN proteomics model achieved high discrimination for pulmonary embolism events in a general population over a long 15-year time horizon. Proteins contributing to the risk are involved in processes relevant to disease pathogenesis, including coagulation (VIT, SPINK6), endothelial function (ENTPD5, ISM1, LYPD3), inflammation (LGALS4), and extracellular matrix remodelling (TNR). Previous efforts in have largely focused on risk prediction in individuals with established disease such as venous thromboembolism^12^. There are also several models with simple clinical variables derived from electronic health record data which predict risk of future venous thromboembolism which often precedes pulmonary embolism^13^. Our work highlights the potential utility of proteomic risk scores in capturing complex, multidimensional biological signatures associated with pulmonary embolism events as well as their potential utility to identify patients at risk of severe thrombotic events several years before disease onset and guide prophylactic treatment or help avoid medication which might increase the risk of pulmonary embolism^14,15^.

Through this systematic analysis, we demonstrate the potential to identify core sets of proteins that could significantly improve the prediction of multiple diseases. Importantly, we show that the optimal method for selecting these proteins—whether linear models or neural networks—varies depending on the specific disease and its unique prediction requirements. Beyond the examples of Parkinson’s disease and pulmonary embolism discussed earlier, our findings underscore the broader promise of proteomic risk scores in predicting several other common outcomes. However, we present this as a proof-of-concept study. A comprehensive evaluation of prediction algorithms for each selected outcome would require focused efforts, including model calibration, detailed assessment of existing or newly developed models for each disease, and decision analyses tailored to different treatment or screening thresholds^2^. Such work extends beyond the scope of this study and should be customized to address the specific needs of individual outcomes.

The examination of shared and unique proteins across a range of diseases provides valuable insights into the overlapping and distinct risk factors among various conditions. Consistent with prior findings, we identified several proteins that act as shared predictors across multiple diseases. These shared proteins likely represent systemic pathways central to the development of multimorbidity, including processes such as aging, stress response, and inflammation. For instance, BCAN (brevican) was inversely associated with the risk of several conditions. As a key extracellular matrix protein in the central nervous system^16^BCAN may also indicate systemic effects or reflect resilience to a broad spectrum of pathological processes. At the same time, we identified numerous unique predictors that may be specific to particular outcomes or offer limited additional value to already selected markers. While these models hold promise for improving prediction accuracy, it is important to note that their use is not intended to draw causal inferences.

Several limitations should be acknowledged. Disease misclassification may have occurred, as we relied solely on hospital episode statistics and did not have access to other sources such as cancer registry data, potentially leading to incomplete case identification. Additionally, our definition of prevalent cases was based solely on retrospective electronic health records, without incorporating self-reported diagnoses, which may have resulted in the omission of a small number of additional prevalent cases. The 5 years follow up analyses has substantially smaller number of incident events compared to the 15 years analyses, which limits the statistical power of models investigating this time horizon and may cause unreliable estimates. Other limitations of our work include the need for validation of the models in independent external populations, the limitation of our analyses on outcomes that have sufficient sample size within the UK biobank sub cohort, the restriction to the proteins measured in the OLINK panel used in UK biobank and the lack of generalisation of our findings to other ethnically diverse populations. Finally, while we employed NN models, their inherently complex nature limits interpretability, as we cannot precisely determine the nonlinear effects and interactions within the models.

In conclusion, proteomics risk scores, either linear or NN models, demonstrated strong discriminatory ability for a variety of other outcomes over both short- and long-term follow-up periods. These outcomes span diseases for which established prediction models already exist such as CHD, as well as conditions that currently lack robust predictive tools. For diseases with existing models, proteomics-based approaches provide an opportunity to enhance accuracy by incorporating easily measured predictors not captured by traditional clinical factors. For diseases without current models, these scores offer a promising avenue for early detection and risk stratification. Overall, proteomic scores show potential in bridging gaps in predictive healthcare, fostering targeted interventions, and supporting precision medicine initiatives aimed at improving outcomes across a wide spectrum of diseases.

## Methods

### Study overview

We used data from UK Biobank (UKB), a general population cohort study, which enrolled 502,536 volunteers aged 40 to 69 years from 2006 to 2010 in 22 recruitment centres across the United Kingdom. Proteomic profiling of blood plasma samples collected at participant recruitment was conducted on a randomised subset of individuals using the Olink platform (*N* = 53,030) in stored serum samples as previously described^17^. For all participants, retrospective and prospective linkage to electronic health data was available, including on-going primary (readcode) data, hospital episode statistics data on hospital admissions, and Office for National Statistics cause of death data. We selected outcomes with more than 100 number of incident cases based on 15-year follow-up which could be captured through electronic health record focusing on common chronic diseases including cardiometabolic, respiratory, mental health and neurological outcomes as well as common cancers. Overall, 27 different outcomes were examined and were classified by the CALIBER code list^18^patients with prevalent disease were excluded for each endpoint (Supplementary Tables 9& Supplementary Table 10). Disease cases were identified using readcode and ICD-10 codes within the June 2023 release of participant linked general practitioner records, hospital episode statistics and death records. The study population was restricted to UKB individuals with proteomic data and divided into a training and test set on a per outcome basis (see statistical analysis section for details). In these sets, we examined the predictive performance of proteomic risk scores, of a basic clinical risk score and their combination. The clinical risk score included cardiovascular predictors used in the pooled cohort’s equation from the American Heart Association^19^ as well as ethnicity and education which are associated with a vast number of outcomes. The test set samples were used for model evaluation in survival analysis models. The performance contribution of a linear and a more complex non-linear NN proteomic risk score were compared and the important proteins for each risk score were identified.

Townsend deprivation index was available for all participants corresponding to the census output area in which their residential postcode is located. Total serum cholesterol, HDL cholesterol and HbA1c levels were obtained from enzymatic assays (Backman Coulter AU5800). We calculated mean systolic blood pressure from two measurements taken seated after two minutes rest using an appropriate cuff and an Omron HEM-7015IT digital BP monitor or manual reading. To define treated hypertension, we used information from self-administrated questionnaire on blood pressure lowering medication.

### Statistical analysis

#### Proteomic risk score calculation

We excluded individuals with > 25% missing values across all proteins (*N* = 8,499) and excluded proteins with > 25% missing values across all remaining samples (*N* = 4). All remaining missing values were imputed using K-Nearest Neighbours. Two different approaches to calculate a univariate proteomic risk scores for each outcome based on 2,919 measured proteins were used, a linear ElasticNet regression model (linear model) and a non-linear NN model, separately for each outcome using all cases within a 15-year follow period. For each outcome, an outcome specific control set was created using all UK BioBank individuals that did not have an incident or prevalent diagnosis of the outcome examined within 15-years follow-up. To allow for unbiased estimation of model performance, a held-out test set was created for each endpoint and kept identical for the linear and neural network risk models. Random stratified sampling of the incidence cases separately for each outcome was applied to ensure an equal split of case/controls in the training and test sets with an 80/20 split ratio (see Table 1 for sample sizes corresponding to each outcome). Hyperparameter tuning was performed for each model using the training set with fivefold cross-validation and all data was standardised using estimates collected from the training set within each fold. Final models were trained using the entire training set with optimal hyperparameters and evaluated using the test set (see Supplementary Tables 2 & Supplementary Table 8 for full hyperparameters details).

Linear risk scores were generated using ElasticNet regression models with standardised protein levels as exposure and each disease as outcome. Model performance was evaluated primarily using area under the receiver operating curve (AUROC), in combination with mean squared error and area under the precision recall curve (AUPRC) which were checked for consistency. NN risk scores were generated using a feed-forward neural network with the same exposures and outcomes. To improve the classification performance, the NN was pre-trained using unsupervised contrastive learning followed by supervised fine-tuning. The contrastive learning was performed using a self-supervised contrastive learning using random feature corruption (SCARF)^20^which applies random feature corruption to generate augmented views of the input data, helping the model learn representations that are robust to noise and distortions. By doing so, the model becomes better at distinguishing between positive pairs—corrupted and original versions of the same instance—and negative pairs from different instances, leading to more discriminative features. Self-distillation was performed by initially training a teacher model using the contrastive learning pre-trained model attached to a disease classification head. We subsequently trained a student model with the same model architecture using the teacher to provide soft labels for self-distillation^21^. To address the large class imbalance issue with deep learning models, we explored the application of Synthetic Minority Oversampling Technique (SMOTE)^22^under sampling of the majority (control) class and class-weighted loss to the training set during the hyperparameter tuning stage for each outcome. Model performance was evaluated primarily using AUROC, in addition to checking for consistency using Brier loss for the positive class and AUPRC. When the addition to a basic clinical risk score of the non-linear risk score compared to the linear risk score provided greater than a 0.05 C-Index improvement in the 15 years follow up model, we calculated the protein contribution to the non-linear risk score using Shapley additive explanation (SHAP) values^23^. SHAP values provide individual level protein importance to risk score generation which allows for the identification of important proteins for subpopulations. However, we are interested in the proteins important for the majority of the population. To identify the globally important proteins, we filtered out the proteins with non-zero SHAP values in less than 25% of the samples for each outcome.

Survival analysis was conducted using Cox ElasticNet models for each outcome utilising the same outcome specific training and held-out test set splits as the risk score generation pipeline. Our primary analysis used a prediction horizon of 15 years of follow up (maximum follow up time in UKB). We also performed secondary analysis with 5 years of follow up to examine the value of the proteomic scores in short term diagnoses of common chronic diseases. Participant follow-up started at the date of their blood sample plasma collection, equivalent to their first visit to a UKB recruitment centre. Time-to-event was set at whichever occurred first; the first instance of disease diagnosis, death of the participant or censoring date (June 2023). For each disease outcome, participants with the first instance of disease diagnosis before the collection of their blood plasma sample were excluded. For type-2 diabetes, we additionally excluded individuals with HbA1c > 6.5% at baseline. Three models were explored per outcome: a model with only clinical factors (age, sex, ethnicity, deprivation index, systolic blood pressure, blood pressure medication usage, HDL cholesterol, total cholesterol and HbA1c), a model with clinical factors plus the univariate linear proteomic risk score for each outcome and a model with clinical factors plus the univariate non-linear proteomic risk score for each outcome. To ensure unbiased estimation of model performance, model discrimination was evaluated using Uno’s C-index^24^ on the held-out test set. We defined the linear and NN proteomic risk scores as equivalent if the C-index between the two models was smaller than 0.02 (ΔC index < 0.02).

### Software

All analysis was performed using Python v3.7.9. Data processing, cross-validation and ElasticNet regressions were performed using the sci-kit learn package^25^. Neural network models were created using the pytorch package^26^. Survival analysis and evaluation were performed using the sksurv package^27^.

## Electronic supplementary material

Below is the link to the electronic supplementary material.

Supplementary Material 1

Supplementary Material 2

Supplementary Material 3

## Data Availability

All proteomic, covariate and disease outcome data used in this study are available from UKB to bona fide researchers upon successful application (https://www.ukbiobank.ac.uk).
