# Supplementary material for: Proteomic risk scores for predicting common diseases using linear and neural network models in the UK biobank
Source: Sci Rep. 2025 Jul 1;15:20520. doi: 10.1038/s41598-025-06232-1 (PMC12219849; doi:10.1038/s41598-025-06232-1)

**Extended Data Figure 1**. Survival analysis performance across all diseases for 5-years follow-up. Yellow bars indicate predictive superiority using the linear risk score, green for the neural network risk score.
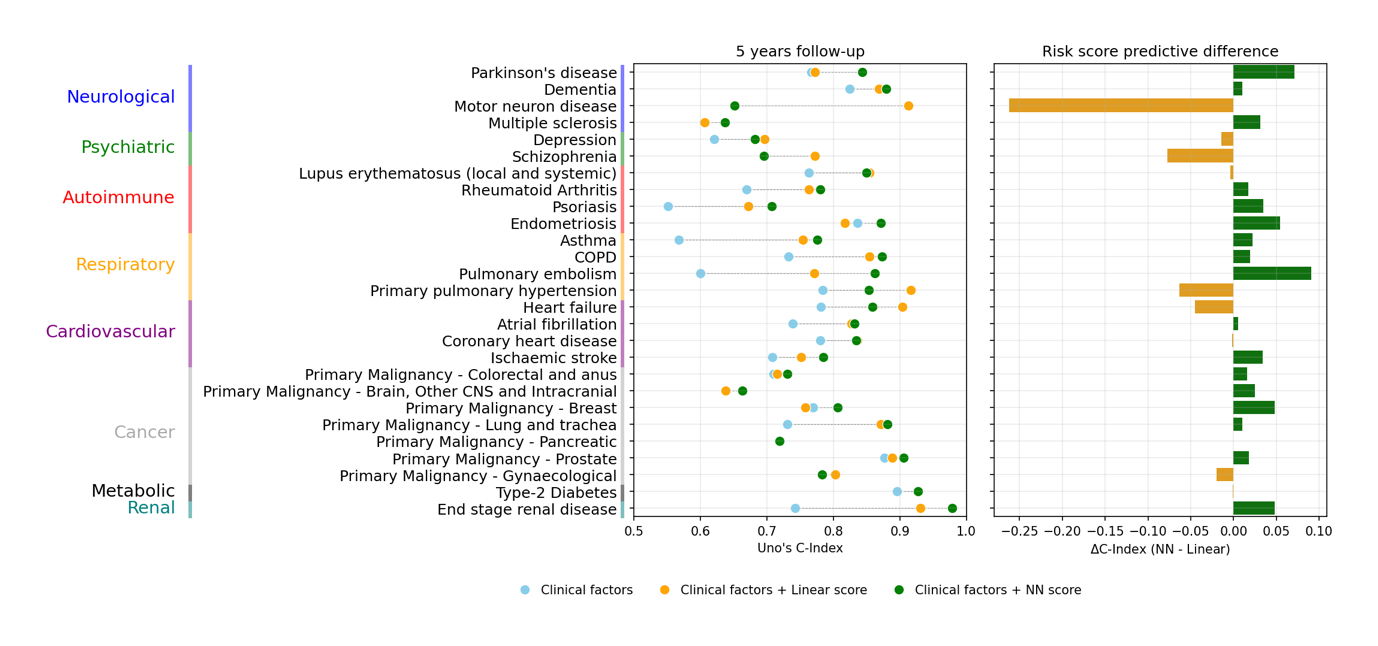


**Extended Data Figure 2.** Bee swarm plots for protein SHAP values for end-stage renal disease (A) and lupus erythematosus (B).

A


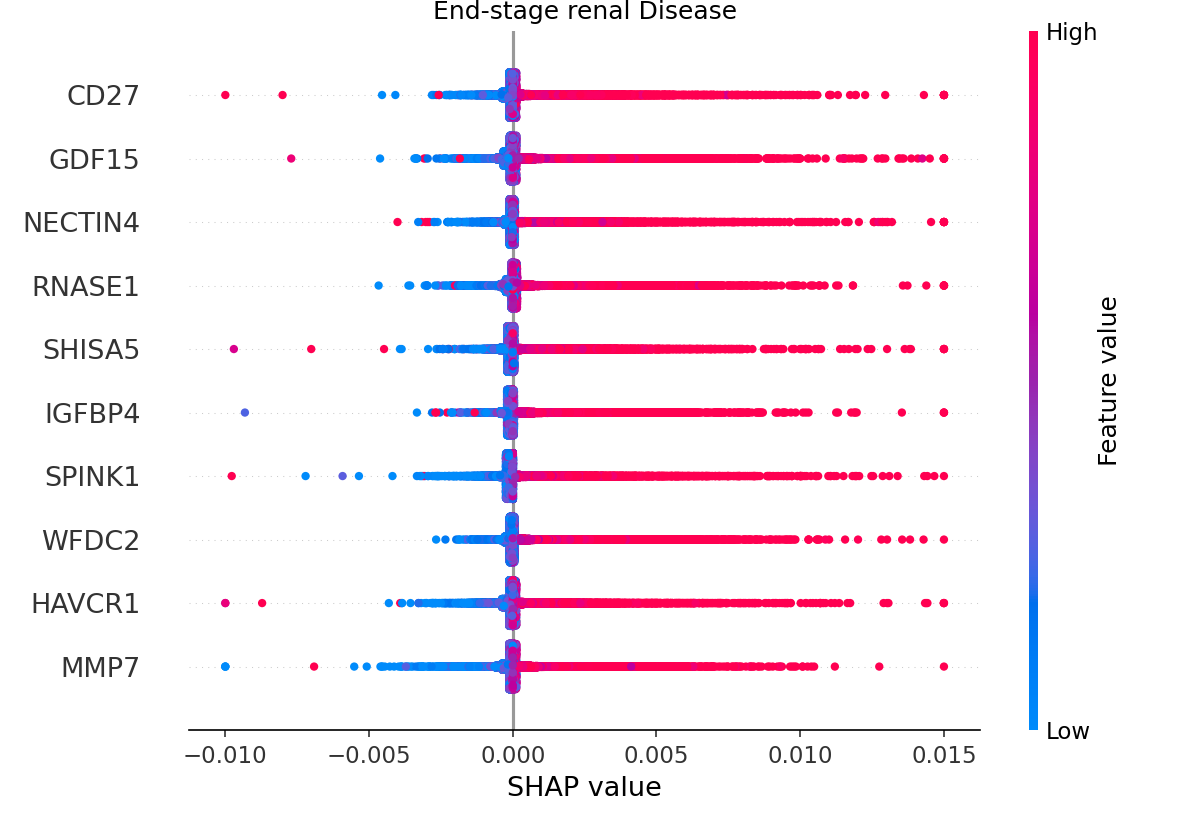


B


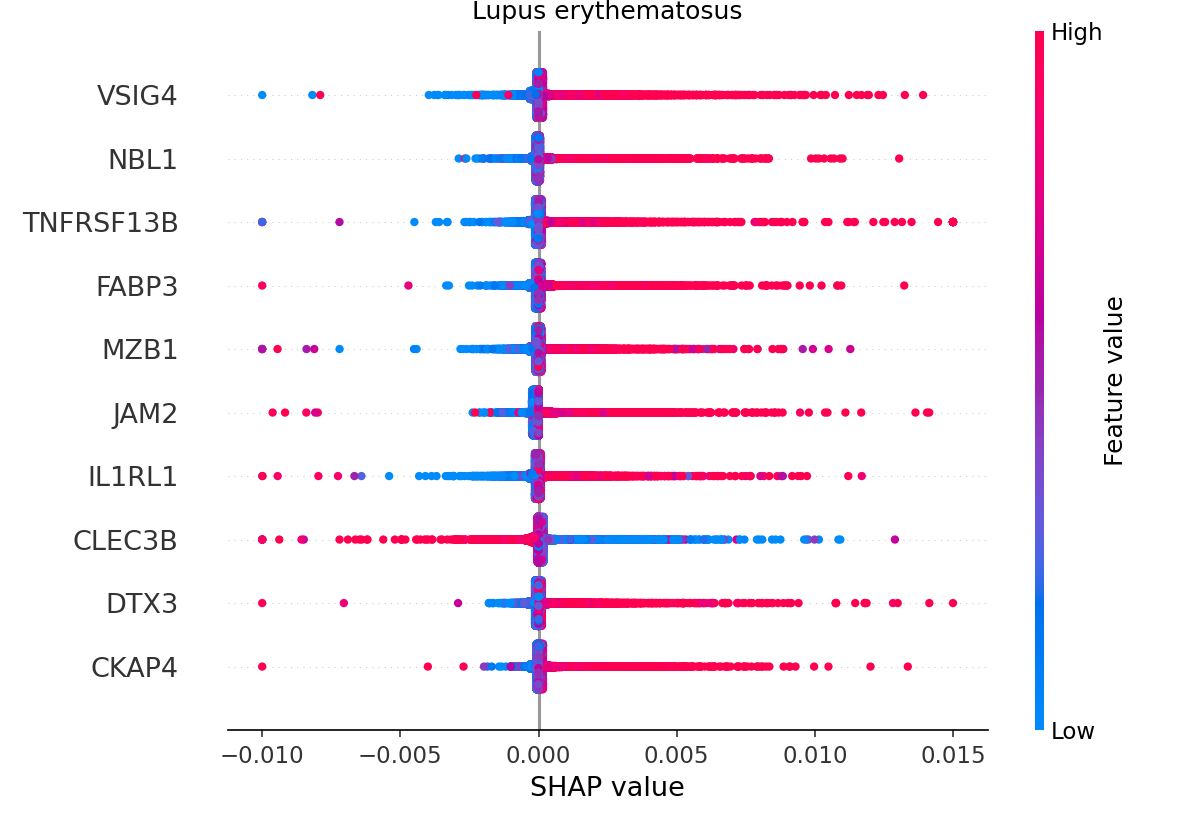

Supplement: Supplementary file 1 — Supplementary Material 1 [file 41598_2025_6232_MOESM1_ESM.docx]
