# Supplementary material for: Proteomic risk scores for predicting common diseases using linear and neural network models in the UK biobank
Source: Sci Rep. 2025 Jul 1;15:20520. doi: 10.1038/s41598-025-06232-1 (PMC12219849; doi:10.1038/s41598-025-06232-1)
Supplement: Supplementary file 3 — Supplementary Material 3 [file 41598_2025_6232_MOESM3_ESM.docx]

**Supplementary Table Legends**

**Supplementary Table 1.** Characteristic table for the UK BioBank participants used in the study.

**Supplementary Table 2.** Hyperparameters of the student & teacher models for the non-linear risk score generation.

**Supplementary Table 3.** Survival analysis results for 15-year follow up

**Supplementary Table 4.** Survival analysis results for 5-year follow up

**Supplementary Table 5.** Standardised beta co-efficient’s for all protein contributions to linear risk scores

**Supplementary Table 6.** Summary of protein contributions to the linear protein risk scores

**Supplementary Table 7.** SHAP values for outcomes where the non-linear risk score outperformed (>0.05 C-Index) the linear risk scores.

**Supplementary Table 8.** The table of hyperparameters and their respective values which were explored during hyperparameter tuning for each outcome.

**Supplementary Table 9.** Readcode codelists for each outcome explored in the study based on CALIBER codelists.

**Supplementary Table 10.** ICD-10 codelists for each outcome explored in the study based on CALIBER codelists.
